# The Enzymatic Core of the Parkinson’s Disease-Associated Protein LRRK2 Impairs Mitochondrial Biogenesis in Aging Yeast

**DOI:** 10.3389/fnmol.2018.00205

**Published:** 2018-06-21

**Authors:** Andreas Aufschnaiter, Verena Kohler, Corvin Walter, Sergi Tosal-Castano, Lukas Habernig, Heimo Wolinski, Walter Keller, F.-Nora Vögtle, Sabrina Büttner

**Affiliations:** ^1^Institute of Molecular Biosciences, University of Graz, Graz, Austria; ^2^Institute of Biochemistry and Molecular Biology, ZBMZ, Faculty of Medicine, University of Freiburg, Freiburg, Germany; ^3^Faculty of Biology, University of Freiburg, Freiburg, Germany; ^4^Department of Molecular Biosciences, The Wenner-Gren Institute, Stockholm University, Stockholm, Sweden

**Keywords:** LRRK2, Parkinson’s disease, neurodegeneration, mitochondria, complex IV, cell death, yeast, aging

## Abstract

Mitochondrial dysfunction is a prominent trait of cellular decline during aging and intimately linked to neuronal degeneration during Parkinson’s disease (PD). Various proteins associated with PD have been shown to differentially impact mitochondrial dynamics, quality control and function, including the leucine-rich repeat kinase 2 (LRRK2). Here, we demonstrate that high levels of the enzymatic core of human LRRK2, harboring GTPase as well as kinase activity, decreases mitochondrial mass via an impairment of mitochondrial biogenesis in aging yeast. We link mitochondrial depletion to a global downregulation of mitochondria-related gene transcripts and show that this catalytic core of LRRK2 localizes to mitochondria and selectively compromises respiratory chain complex IV formation. With progressing cellular age, this culminates in dissipation of mitochondrial transmembrane potential, decreased respiratory capacity, ATP depletion and generation of reactive oxygen species. Ultimately, the collapse of the mitochondrial network results in cell death. A point mutation in LRRK2 that increases the intrinsic GTPase activity diminishes mitochondrial impairment and consequently provides cytoprotection. In sum, we report that a downregulation of mitochondrial biogenesis rather than excessive degradation of mitochondria underlies the reduction of mitochondrial abundance induced by the enzymatic core of LRRK2 in aging yeast cells. Thus, our data provide a novel perspective for deciphering the causative mechanisms of LRRK2-associated PD pathology.

## Introduction

Parkinson’s disease (PD) is a neurodegenerative disorder of multifactorial etiology with genetics, environmental toxins and age as determinant risk factors. While the majority of PD cases are sporadic, various monogenic forms of PD have been identified (Thomas and Beal, 2007; Klein and Westenberger, 2012; Hernandez et al., 2016). Accumulating evidence points towards shared cellular pathways driving sporadic and familial PD pathology (Klein and Westenberger, 2012). Consistently, several genes causative for familial PD have also been shown to constitute risk factors for the development of sporadic PD. Importantly, impairment of mitochondrial function is a reoccurring phenotype in PD. Samples derived from sporadic PD patients display respiratory chain complex I deficiency (Schapira et al., 1990; Parker et al., 2008). Similarly, mutations in genes associated with both autosomal recessive and dominant familial PD interfere with mitochondrial function, in particular mitochondrial quality control, at different levels and in diverse model systems (Larsen et al., 2018). Thus, unraveling the link between PD and mitochondrial dysfunction is fundamental to understand disease development.

The most prevalent genetic cause of both familial and sporadic PD is linked to sequence variations in the gene encoding the leucine-rich repeat kinase 2 (LRRK2; Klein and Westenberger, 2012; Hernandez et al., 2016). LRRK2 has been proposed to function in a wide variety of cellular processes, including vesicle trafficking and endocytosis, autophagy, regulation of the retromer complex as well as mitochondrial function and, in turn, dysregulation of these processes is reported for diverse PD-associated LRRK2 mutations (Wallings et al., 2015). Still, the molecular mechanisms of its physiological and pathological mode of action are mostly unknown. LRRK2 is a large, multifunctional protein with several domains, including distinct protein-protein interaction domains and an enzymatic core composed of a Ras-of-complex (ROC) GTPase, an associated C-terminal-of-ROC (COR) domain and a serine/threonine protein kinase (Islam and Moore, 2017). The most frequent pathogenic mutations in LRRK2, such as R1441C/G/H, Y1699C, G2019S and I2020T, are located within its catalytic core and affect its enzymatic activity. The GTPase and the kinase activity mutually influence each other, but their interplay as well as their specific contribution to neurotoxicity are not fully understood yet. Whereas kinase hyperactivation (G2019S and I2020T) as well as decreased activity of the GTPase (R1441C/G/H and Y1699C) are linked to PD, enhanced GTPase activity as seen for the synthetic R1398L mutant or the R1398H variant in humans reduce toxic consequences mediated by LRRK2 or the risk for onset of PD, respectively (Chen et al., 2011; Ross et al., 2011; Heckman et al., 2014; Nguyen and Moore, 2017; Ramírez et al., 2017).

Several *in vivo* and *in vitro* models display distinct mitochondrial abnormalities upon high levels of LRRK2 variants, but the effects of particular point mutations on the observed mitochondrial changes remain controversial (Mortiboys et al., 2010; Cooper et al., 2012; Godena et al., 2014; Thomas et al., 2016; Schwab et al., 2017). Common phenotypes observed in most studies include a decrease in mitochondrial transmembrane potential, alterations of mitochondrial trafficking and a depletion of mitochondria, specifically within neurites. This reduction of mitochondrial mass has been attributed to either increased retrograde trafficking of mitochondria (Schwab et al., 2017), to inhibition of both anterograde and retrograde trafficking (Godena et al., 2014; Thomas et al., 2016), or to excessive mitochondrial degradation via autophagy (Cherra et al., 2013). Other studies observed no effect on mitochondrial content but a dissipation of mitochondrial transmembrane potential, resulting in a depletion of cellular ATP levels (Mortiboys et al., 2010; Papkovskaia et al., 2012). Thus, the effects of LRRK2 on mitochondrial function seem to be rather pleiotropic. Interestingly, genetically enforcing mitochondrial biogenesis has been recently shown to alleviate LRRK2-induced degeneration in a *Drosophila* model for PD (Ng et al., 2017). Yet it has not been analyzed whether defects in mitochondrial biogenesis represent a causative factor for the LRRK2-triggered decrease in mitochondrial mass.

Here, we establish an aging yeast model for LRRK2 cytotoxicity and show that expression of the enzymatic core of LRRK2, consisting of its ROC, COR and kinase domain, hereafter referred to as LRRK2^RCK^, decreases mitochondrial mass via an impairment of mitochondrial biogenesis prior to the induction of age-dependent cell death. We link mitochondrial depletion to a specific downregulation of mitochondria-related gene transcripts and identify complex IV deficiency as early event in LRRK2^RCK^-triggered cellular demise.

## Materials and Methods

### *Saccharomyces cerevisiae* Strains and Genetics

All experiments were performed in *Saccharomyces cerevisiae* BY4741 (MAT**a**; *his3*Δ1; *leu2*Δ0; *met15*Δ0; *ura3*Δ0) obtained from Euroscarf and experiments shown in Figures 3G–I were additionally performed in *S. cerevisiae* W303 (MAT**a**; *leu2–3,112*;* trp1–1*;* can1–100*;* ura3–1*;* ade2–1*). Plasmids for heterologous expression of the enzymatic core of human wild type LRRK2 (LRRK2^RCK^), the R1389L^RCK^ mutant form with enhanced GTPase activity, and the G2019S^RCK^ variant with increased kinase activity were kindly provided by Darren J. Moore (Xiong et al., 2010). ß-galactosidase (LacZ) expressed from the same plasmid was used as a control. LRRK2^RCK^ variants and LacZ harbored a C-terminal V5 tag and were expressed under the control of a GAL1 promoter from a pYES2/CT plasmid. C-terminally mCherry-tagged versions of these proteins were constructed by PCR-amplification of mCherry, restriction digest with *Xba*I and *Pme*I and ligation into the existing pYES2/CT plasmids encoding the LRRK2^RCK^ variants. mCherry expressed from a pYES2/CT plasmid was used as a control for microscopic analyses, and was cloned by PCR amplification of mCherry, restriction digest with *Bam*HI and *Not*I and ligation into the pYES2/CT plasmid. All oligonucleotides used in this study are listed in Supplementary Table S1. Standard lithium acetate method was used for yeast transformation (Gietz and Woods, 2002). Genomically modified strains were created by homologous recombination as previously described (Janke et al., 2004). All strains used in this study are listed in Supplementary Table S2. After plasmid transformation or genomic modification, at least four different clones were tested to rule out clonogenic variations.

**Figure 1:**
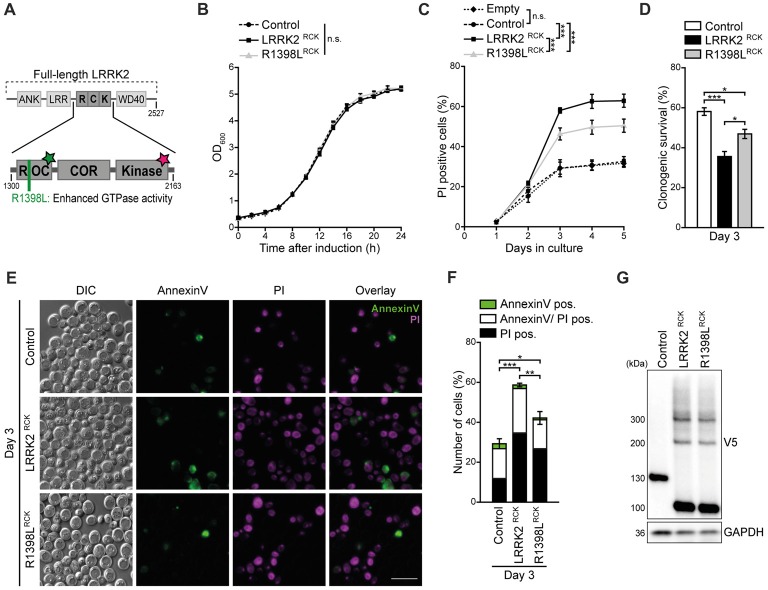
LRRK2^RCK^ triggers death of aging yeast cells. **(A)** Scheme of truncated leucine-rich repeat kinase 2 (LRRK2) constructs used in this study. The enzymatic core of human LRRK2 (amino acids 1300–2163) containing the Ras-of-complex (ROC) GTPase, the C-terminal-of-ROC (COR) and the protein kinase domain, was expressed in yeast cells during chronological aging under the control of a GAL1 promoter. The wild type form of truncated LRRK2 (hereinafter referred as LRRK2^RCK^) and the point mutant R1398L^RCK^ with higher GTPase activity were used. The green star indicates GTPase activity, the star in magenta represents kinase activity. **(B)** Growth of control cells expressing LacZ compared to cells expressing LRRK2^RCK^ and the R1398L^RCK^ variant. OD_600_ was measured in intervals of 2 h starting from the induction of galactose-driven expression. Means ± SEM; *n* = 4. **(C)** Flow cytometric quantification of loss of membrane integrity as indicated with propidium iodide (PI) staining of cells as described in **(B)**. In addition, cells harboring the empty vector were analyzed to validate the suitability of LacZ expression as a control. Significances represent simple main effects between different expression types at each time point. Significances shown are valid for day 3–5. Means ± SEM; *n* = 4. **(D)** Clonogenic survival on day 3 of chronological aging determined by counting colony forming units (cfu) after plating 500 cells with indicated expression types on YEPD agar plates. Means ± SEM; *n* = 8. **(E,F)** AnnexinV/PI co-staining on day 3 of aging. Representative epifluorescence micrographs **(E)** and flow cytometric quantification **(F)** are shown. Scale bar represents 10 μm. Means ± SEM; *n* = 4. For AnnexinV/PI staining at earlier time points, please see Supplementary Figures S1D,E. **(G)** Immunoblot analysis of protein extracts from cells as described in **(B)**. Blots were probed with antibodies directed against the V5-epitope to detect V5-tagged LacZ, LRRK2^RCK^ and R1398L^RCK^, and against glyceraldehyde 3-phosphate dehydrogenase (GAPDH) as a loading control. ***p < 0.001, **p < 0.01, *p < 0.05, n.s. not significant.

**Figure 2:**
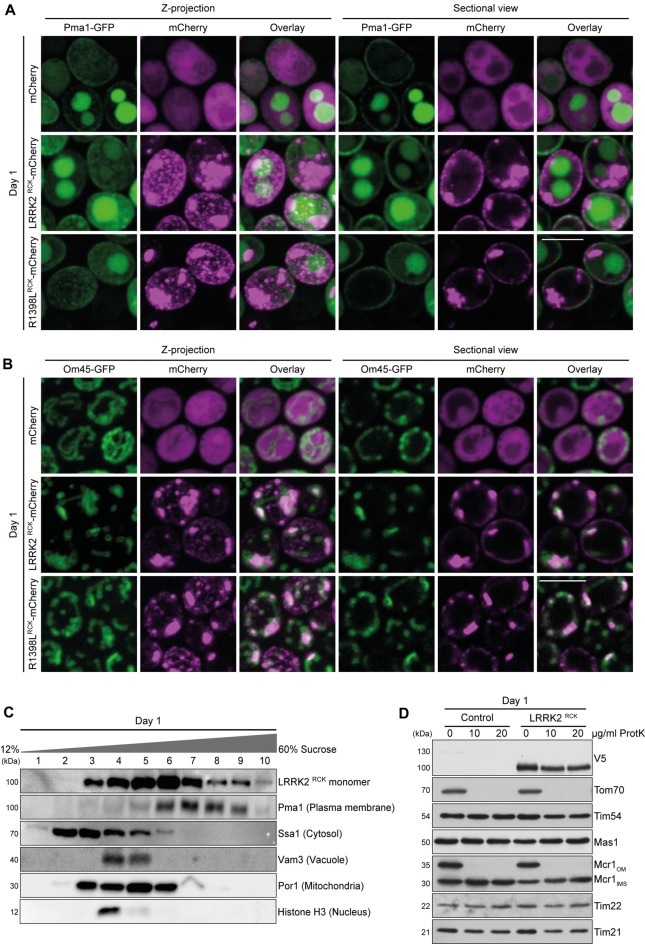
LRRK2^RCK^ localizes in mitochondria and at the plasma membrane. **(A)** Representative confocal micrographs of strains harboring endogenously green fluorescent protein (GFP)-tagged Pma1 expressing mCherry alone or fused to LRRK2^RCK^ and R1398L^RCK^ on day 1 of aging. Z-projections of three-dimensional stacks as well as a representative section are shown. Scale bar represents 5 μm. For quantification of colocalization and confocal micrographs of strains harboring GFP-tagged Htb2, please see Supplementary Figures S2A,B. **(B)** Representative confocal micrographs of strains harboring endogenously GFP-tagged Om45, expressing the constructs as described in **(A)**. Z-projections of three-dimensional stacks as well as a representative section are shown. Scale bar represents 5 μm. For quantification of colocalization please see Supplementary Figure S2B. **(C)** Representative immunoblots of subcellular fractionation of whole cell extracts on 12%–60% step sucrose gradients. Collected fractions were analyzed by immunoblotting. Blots were probed with antibodies directed against the V5-epitope to detect V5-tagged LRRK2^RCK^, and against organelle-specific marker proteins as indicated. **(D)** Immunoblot analysis of purified mitochondria obtained from cells expressing LRRK2^RCK^ or LacZ on day 1. Prior to SDS-PAGE, samples were treated with depicted concentrations of proteinase K (ProtK). Blots were probed with antibodies directed against the V5-epitope, the outer mitochondrial membrane protein Tom70, the inner mitochondrial membrane proteins Tim54, Tim22 and Tim21, the mitochondrial matrix localized protease Mas1, as well as Mcr1, located in the outer membrane (OM) and intermembrane space (IMS).

**Figure 3:**
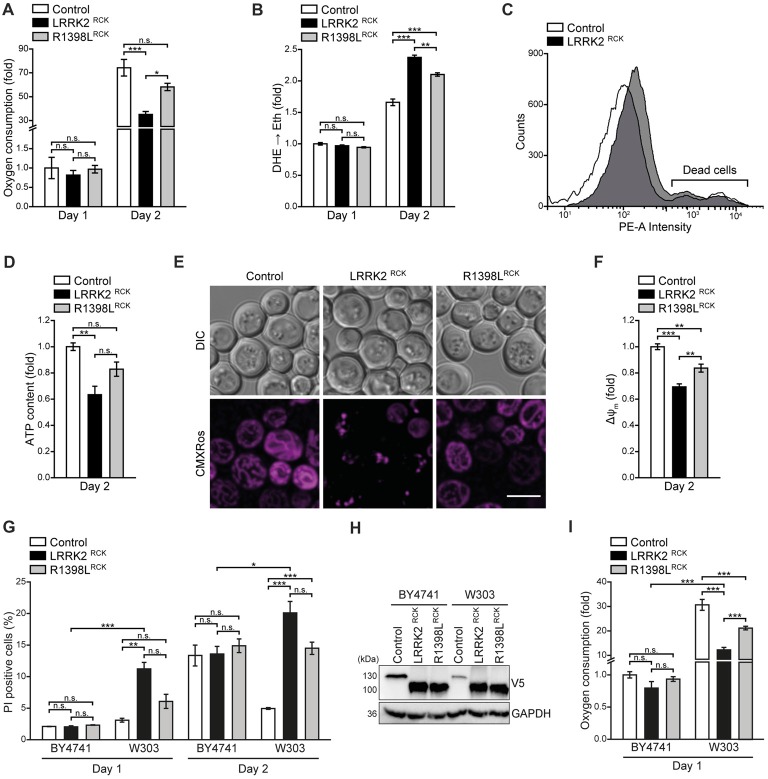
LRRK2^RCK^ impairs mitochondrial function. **(A)** Oxygen consumption determined with a Fire-Sting optical oxygen sensor system in cells expressing LacZ, LRRK2^RCK^ or R1398L^RCK^ on day 1 and 2 of aging. Oxygen consumption was normalized to living cells and subsequently depicted as fold of control cells. Means ± SEM; *n* = 4. **(B,C)** Flow cytometric quantification of the reactive oxygen species (ROS)-driven conversion of non-fluorescent dihydroethidium (DHE) to fluorescent ethidium (Eth) in cells described in **(A)**. Mean fluorescence intensities are shown as fold of control cells on day 1 **(B)**. Dead cells, which accumulated Eth due to a loss of membrane integrity, were excluded from the analysis as shown in **(C)**. Means ± SEM; *n* = 4. **(D)** Quantification of ATP content from cells described in **(A)** on day 2 of aging. Values were normalized to living cells and subsequently depicted as fold of control cells. Means ± SEM; *n* = 4. **(E,F)** Analysis of the mitochondrial transmembrane potential (ΔΨ_m_) with Mitotracker CMXRos on day 2 of aging. Representative confocal micrographs **(E)** and flow cytometric quantification of the mean fluorescence intensity **(F)** are shown. Values for ΔΨ_m_ were normalized to control cells. Scale bar represents 5 μm. Means ± SEM; *n* = 4. **(G)** Flow cytometric quantification of loss of membrane integrity as indicated with PI staining of BY4741 and W303 strains expressing LacZ, LRRK2^RCK^ or R1398L^RCK^ on day 1 and day 2 of aging. Means ± SEM; *n* = 4. **(H)** Immunoblot analysis of protein extracts from cells as described in **(G)**. Blots were probed with antibodies directed against the V5-epitope and against GAPDH as loading control. **(I)** Oxygen consumption determined with a Fire-Sting optical oxygen sensor system in cells as described in **(G)** on day 1 of aging experiments. Normalization was performed as described in **(A)**. Means ± SEM; *n* = 4. ***p < 0.001, **p < 0.01, *p < 0.05, n.s. not significant.

### Media and Culturing Conditions

All strains were grown at 28°C and 145 rpm in synthetic complete (SC) medium, consisting of 0.17% yeast nitrogen base (Difco, BD Biosciences), 0.5% (NH_4_)_2_SO_4_ and 30 mg/l of all amino acids (except 80 mg/l histidine and 200 mg/l leucine) and 30 mg/l adenine with 2% D-glucose (SCD), or 2% D-galactose (SCG) for GAL1-driven expression of LacZ and LRRK2^RCK^ variants. Media were prepared using double distilled water and autoclaved for 25 min at 121°C, 210 kPa. Amino acids were sterilized separately as 10× stocks and admixed to the media after autoclaving. Full media (YEPD) contained 1% yeast extract (Bacto, BD Biosciences), 2% peptone (Bacto, BD Biosciences) and 4% D-glucose. For solid media, 2% agar was admixed and hygromycin (300 mg/l), nourseothricin (100 mg/l) or geneticin (300 mg/l) was added after autoclaving when required to select for transformants.

For chronological aging experiments, overnight cultures grown in SCD media for 16–20 h were used to inoculate 10 ml SCD in 100 ml baffled Erlenmeyer flasks to an OD_600_ of 0.1. Cells were further grown to an OD_600_ of 0.3, collected by centrifugation (5 min, 2300 rcf), and resuspended in 10 ml SCG media to induce GAL1-dependent expression. Aliquots were taken at indicated time points to study survival and mitochondrial abundance, morphology and function. To analyze exponential growth after induction of galactose-driven expression, OD_600_ was measured every 2 h for 24 h on a Beckman coulter DU 730 Life Science UV/VIS Spectrophotometer.

### Analysis of Cell Death

Loss of membrane integrity was determined with propidium iodide (PI) staining as previously described (Aufschnaiter et al., 2017). In brief, 2*10^6^ cells were harvested in 96-well plates at indicated time points and resuspended in 250 μl of phosphate buffered saline (PBS, 25 mM potassium phosphate, 0.9% NaCl; adjusted to pH 7.2) containing 100 μg/l PI. After incubation for 10 min at room temperature (RT) in the dark, cells were washed once in 250 μl PBS and subsequently analyzed via flow cytometry (BD LSR Fortessa; 30,000 cells were evaluated with BD FACSDivia software) and/or fluorescence microscopy.

To discriminate between necrotic and early/late apoptotic cell death, AnnexinV/PI co-staining was applied as described previously (Büttner et al., 2007, 2011). 2*10^7^ cells were harvested, washed once in digestion buffer (35 mM K_3_PO_4_, 0.5 mM MgCl_2_, 1.2 M sorbitol; adjusted to pH 6.8) and subsequently resuspended in 330 μl of the same buffer. After addition of 2.5 μl Lyticase (1000 U/ml) and 15 μl Glucoronidase/Arylsulfatase (4.5 U/ml), cells were incubated at 28°C and 145 rpm for about 1 h to digest the cell wall. Spheroplasts were carefully washed in 500 μl digestion buffer, resuspended in 30 μl staining buffer (10 mM HEPES, 140 mM NaCl, 5 mM CaCl_2_, 0.6 M sorbitol; adjusted to pH 7.4) containing PI and AnnexinV-FITC (Roche) and incubated for 20 min at RT in the dark. 250 μl staining buffer were added to each sample and cells were evaluated via flow cytometry and fluorescence microscopy.

To measure clonogenic survival, the cell number of each culture was determined with a CASY cell counting device (Schärfe Systems) and 500 cells were plated on agar plates with YEPD media. Plates were incubated at 28°C for 2 days and colony forming units (cfu) were analyzed with Fiji software (Schindelin et al., 2012).

### Measurement of Oxidative Stress and Mitochondrial Transmembrane Potential in Whole Cells

Oxidative stress was monitored via the reactive oxygen species (ROS) driven conversion of non-fluorescent dihydroethidium (DHE) to fluorescent ethidium (Eth; Büttner et al., 2007). Briefly, 2*10^6^ cells were harvested in 96-well plates at indicated time points. Cells were then resuspended in 250 μl of PBS containing 2.5 mg/l DHE and incubated for 5 min at RT in the dark. After washing once with PBS, cells were analyzed via flow cytometry. Of note, dead cells, accumulating the fluorescent dye, were excluded from the analysis (gating strategy is depicted in Figure 3C) and the mean fluorescence intensity of living cells was quantified. Results are presented as fold of control cells expressing LacZ on day one. Mitochondrial transmembrane potential (ΔΨ_m_) was determined with the fluorescent dye Mitotracker CMXRos (Thermo Fisher Scientific). For this purpose, 2*10^6^ cells were harvested and resuspended in 250 μl PBS containing 200 nM Mitotracker CMXRos. After incubation for 10 min at RT in the dark, cells were washed once in PBS and quantified via flow cytometry and analyzed with confocal microscopy.

### Determination of Cellular Oxygen Consumption and ATP Levels

Oxygen consumption of yeast cells was measured with a Fire-Sting optical oxygen sensor system (Pyro Science) at indicated time points. To that end, 2 ml of culture were directly transferred into glass tubes, hermetically sealed and immediately used for the analysis. Oxygen concentration was measured for a period of at least 2 min and the slope of the regression line was calculated. The number of cells was assessed directly from the measurement tubes with a CASY cell counting device and the percentage of dead cells was analyzed via flow cytometric quantification of PI-stained cells as described above. Oxygen consumption per living cell was then calculated, and results were normalized to control cells and depicted as fold values.

To quantify cellular ATP levels, 1 ml of culture was harvested and ATP was extracted with the hot ethanol method. In brief, cells were flash frozen in liquid nitrogen, resuspended in 0.5 ml BES buffer (75% EtOH, 10 mM (NH_4_)_2_SO_4_) and incubated for 3 min at 90°C. Samples were then centrifuged for 20 min at 4°C with 16,100 rcf and diluted 20-fold in Tris buffer (20 mM; pH 8). The ATP content of the extracts was quantified with a luminescent ATP detection assay kit (Abcam). For that, 150 μl of sample were transferred into a low-bind 96-well plate and incubated for 5 min for adaption to RT. 50 μl of supplied substrate solution were added and incubated for 5 min in the dark. The assay plate was placed into a GloMax Multi detection system (Promega) and incubated for further 10 min to allow dark adaption. Luminescence was then measured via integration of the signal over 10 s. The number of living cells was simultaneously quantified as described above to calculate the ATP content of living cells. Results are depicted as fold values via normalization to control cells expressing LacZ.

### Fluorescence Microscopy

Specimen were prepared on agar slides to immobilize yeast cells. Epifluorescence microscopy was performed with a Zeiss Axioskop with appropriate filter settings. For visualization of mitochondrial morphology and colocalization studies, yeast cells were analyzed with a Leica SP5 confocal microscope equipped with a Leica HCX PL APO 63× NA 1.4 oil immersion objective. Green fluorescent protein (GFP) was excited at 488 nm and emission was measured between 500 nm and 530 nm. To exclude dead cells from the analysis of mitochondrial morphology, cells were counterstained with PI as described above, excited at 563 nm and detected between 590 nm and 650 nm. Z-stacks were acquired using 63 × 63 × 125 (x/y/z) nm sampling. Microscopic pictures were analyzed and processed with the open-source software Fiji (Schindelin et al., 2012). To reduce image noise in z-stacks, 3-dimensional Gaussian filtering (xσ = yσ = zσ = 1) was applied, followed by background subtraction (rolling ball radius = 100 pixels). Three-dimensional data was projected using the maximum-intensity projection method. Pictures within an experiment were captured and processed in the same way.

For quantification of colocalization, the Pearson correlation coefficient (PCC) as well as the threshold-corrected Manders’ coefficient M1 (overlap of GFP signal with mCherry-tagged LRRK2^RCK^ variants) and M2 (overlap of mCherry-tagged LRRK2^RCK^ variants with GFP signal) were calculated using the Fiji plugin JACoP (Bolte and Cordelières, 2006). Depicted M1 and M2 coefficients used a threshold value, which was determined with Fiji applying the Mean method. Picture pre-processing was performed as described above. For each strain and expression type, at least 120 cells from three different clones were analyzed (*n* = 3) and means ± SEM are presented.

### Subcellular Fractionation

Subcellular fractionation experiments were adapted from Klasson et al. (1999). Cells equivalent to an OD_600_ of 200 were harvested at day one, flash frozen in liquid nitrogen and stored at −80°C until lysis. Samples were thawed on ice and resuspended in ice-cold water with 10 mM NaN_3_ and centrifuged for 10 min at 500 rcf. Cells were then resuspended in 0.5 ml lysis buffer (10 mM MOPS, 0.8 M sorbitol, 2 mM EDTA, 1 mM phenylmethylsulfonyl fluoride, 1× cOmplete protease inhibitor cocktail (Roche); adjusted to pH 7.2) and approx. 500 μl glass beads (500 μm diameter) were added. Mechanical lysis was performed in three cycles of 1 min. Unlysed cells and cell debris were removed by two centrifugation steps for 5 min at 2400 rcf. The supernatant was carefully loaded on a sucrose step gradient (60%, 54%, 48%, 42%, 36%, 30%, 24%, 18% and 12% (w/v), each 1 ml) and centrifuged for 3 h at 4°C with 150,000 rcf (Beckman coulter LE-80K centrifuge, SW 41 Ti Swinging-Bucket Rotor, 34,000 rpm). Fractions of 1 ml were collected manually from top to bottom, 1 ml 50% trichloroacetic acid was added and incubated for 5 min on ice. After centrifugation (15 min, 16,100 rcf, 4°C), samples were rinsed once in 1 ml of 1 M Tris (untitrated) and resuspended in 150 μl of non-reducing Laemmli buffer (50 mM Tris-HCl, 2% SDS, 10% glycerol, 0.1% bromophenol blue; adjusted to pH 6.8). 50 μl of samples were used for immunoblotting as described below.

### Immunoblotting

Cells equivalent to an OD_600_ of 3 were harvested at indicated time points. For lysis, pellets were resuspended in 200 μl of 0.1 M NaOH and incubated for 5 min at RT with 1400 rpm shaking. Samples were then centrifuged with 1500 rcf for 5 min, resuspended in 150 μl reducing Laemmli buffer (50 mM Tris-HCl, 2% SDS, 10% glycerol, 0.1% bromophenol blue, 100 mM 2-mercaptoethanol; adjusted to pH 6.8) and incubated for 5 min at RT with 1400 rpm shaking. Prior to SDS-PAGE, samples were heated for 10 min to 65°C and centrifuged for 1 min with 16,100 rcf. 15 μl of supernatant was applied for standard SDS-PAGE and immunoblotting was performed using standard protocols. For detection, antibodies against the GFP-epitope (Roche; 11814460001), V5-epitope (Thermo Fisher Scientific; MA5–1525), glyceraldehyde 3-phosphate dehydrogenase (GAPDH; Thermo Fisher Scientific; MA5–15738), Por1 (Thermo Fisher Scientific; 16G9E6BC4) and histone H3 (Abcam; ab1791) were used. Furthermore, antibodies against Mdh1, Tom22, Pma1, Ssa1, Vam3, Sec61, Cox1, Cox3, Cox4, Cox6, Sdh1, Tom70, Tim44, Atp14, Tim54, Mcr1, Tim22, Tim21 and Mas1, which were generated by injecting rabbits with recombinantly expressed and purified proteins, were applied. Peroxidase conjugated secondary antibodies against rabbit (Sigma; A0545) or mouse (Sigma; A9044) were used for chemiluminescence detection.

To analyze LRRK2^RCK^ oligomers, semi-native immunoblots were applied as recently described (Aufschnaiter et al., 2017). Cells equivalent to an OD_600_ of 8 were prepared as described above with non-reducing Laemmli buffer. Polyacrylamide gels without SDS were used for electrophoresis, which was performed at 4°C. For detection of all immunoblots, a ChemiDoc™ Touch Imaging System (Bio-Rad) was used and densitometric quantification was performed with ImageLab 5.2 Software (Bio-Rad). All indicated molecular weights shown in immunoblots represent the apparent molecular weights (kDa), determined with a PageRuler prestained protein ladder (Thermo Fisher Scientific) or a Spectra™ Multicolor High Range Protein Ladder (Thermo Fisher Scientific), as stated by the manufactures migration patterns.

### Quantitative Real-Time PCR

For quantification of mRNA levels, total RNA was isolated from yeast cultures. To that end, cells equivalent to an OD_600_ of 3 were harvested at indicated time points and resuspended in 1 ml of TRIzol reagent (Thermo Fisher Scientific). Approximately 200 μl of glass beads (500 μm diameter) were added and cells were mechanically lysed in three cycles of 1 min. Subsequent steps were performed as described in the manufacturer’s manual. RNA integrity was visualized by agarose-gel electrophoresis (adapted from Aranda et al., 2012). Residual DNA was removed with the DNA-free Kit (Ambion) and the RNA concentration was determined with a NanoDrop 1000 Spectrophotometer. Reverse transcription of 3.5 μg isolated RNA was performed with the FIREScript RT cDNA synthesis kit (Solis Biodyne) using the supplied random hexamer primers. After dilution of the resulting cDNA, q-RT-PCR was performed with the 5× HOT FirePol EvaGreen qPCR Supermix (Solis Biodyne). All primers used for q-RT-PCR are listed in Supplementary Table S1. All primers showed an efficiency between 90% and 110%, as assessed with standard curve experiments. To measure gene expression levels, cDNA was utilized in duplicate for q-RT-PCR and the relative gene expression levels were calculated with the comparative C_T_ method (Livak and Schmittgen, 2001) with normalization to* UBC6* as housekeeping gene.

### Isolation and Purification of Mitochondria

Mitochondria from *S. cerevisiae* strains were isolated by differential centrifugation (Meisinger et al., 2006). Briefly, yeast cells were grown on SCG medium at 28°C for around 19 h until an OD_600_ of 1.5–2 was reached. The cultures were harvested and afterwards incubated for 20 min in pre-warmed dithiothreitol (DTT) buffer (0.1 M Tris-H_2_SO_4_, 10 mM DTT; adjusted to pH 9.4). After centrifugation, the pellet was resuspended in zymolyase buffer (20 mM potassium phosphate buffer, 1.2 M sorbitol; adjusted to pH 7.4) containing 4 mg zymolyase per gram wet weight of the cells and incubated for 30–45 min to generate spheroplasts. Spheroplasts were washed and resuspended at 7 ml/g cells in ice-cold homogenization buffer (10 mM Tris-HCl, 0.6 M sorbitol, 1 mM EDTA, 1 mM phenylmethylsulfonyl fluoride (PMSF), 0.2% (w/v) bovine serum albumin (BSA); adjusted to pH 7.4) and were broken on ice with 30 strokes using a tight-fitting glass douncer with a Teflon pistil. Cell debris and unbroken cells were removed via two times centrifugation (5 min, 2000 rcf, 4°C). The mitochondrial fraction was isolated by centrifugation (15 min, 16,800 rcf, 4°C) and 25 μl aliquots were immediately snap-frozen in liquid nitrogen. Protein concentration was determined with a Bradford assay (Roti-Quant™, Roth) against bovine γ-globulin standard (Bio-Rad) according to the manufacturer’s instructions.

### Measurement of Mitochondrial Transmembrane Potential in Isolated Mitochondria

Isolated mitochondria were used to measure ΔΨ_m_ via fluorescence reduction. 30 μg of crude mitochondria were resuspended in 3 ml membrane potential buffer (20 mM potassium phosphate buffer, 0.6 M sorbitol, 10 mM MgCl_2_, 0.5 mM EDTA, 5 mM L-malate, 5 mM succinate, 0.1% (w/v) BSA; adjusted to pH 7.2) containing 2 μM 3,3′-dipropylthiadicarbocyanine iodide (DiSC_3_, stored in ethanol). Immediately, the absorption (excitation 622 nm, emission 670 nm) was recorded with a luminescence spectrometer (Aminco Bowman 2, Thermo Electron) until a distribution equilibrium was generated. By adding 1 μM valinomycin the ΔΨ_m_ was disrupted and the total fluorescence was measured. Data were analyzed with FL WinLab (PerkinElmer).

### Blue-Native-PAGE and Mitochondrial ATPase Activity Staining

Mitochondrial protein complexes were investigated by solubilizing 80 μg mitochondria in digitonin buffer (20 mM Tris-HCl, 1% (w/v) digitonin, 10% (v/v) glycerol, 0.1 mM EDTA, 50 mM NaCl, 1 mM PMSF; adjusted to pH 7.4) on ice for 15 min. Afterwards, samples were centrifuged (15 min, 16,100 rcf, 4°C) and the supernatant was analyzed on a 4%–10% gradient Blue-Native-PAGE, followed by immunodecoration, which was performed according to standard protocols. The molecular weight of the standard proteins from the HMW Native Marker Kit (GE Healthcare) is depicted for all Blue-Native blots.

For mitochondrial ATPase activity staining, 80 μg mitochondria were lysed in digitonin buffer and protein complexes were separated using Blue-Native-PAGE. The gel was washed in distilled H_2_O for 20 min and immediately incubated in ATP-buffer (50 mM glycine, 20 mM ATP, 5 mM MgCl_2_; adjusted to pH 8.4 with NaOH) for 20 min. The gel was transferred into a 10% (w/v) CaCl_2_ solution and incubated until calcium phosphate precipitation was visible, washed with distilled H_2_O, before documentation was carried out by using the LAS4000 camera system (Fujifilm).

### Outer Mitochondrial Membrane Integrity Test

40 μg of mitochondria were diluted in 100 μl SEM buffer (10 mM MOPS, 250 mM sucrose, 1 mM EDTA; adjusted to pH 7.2) and treated with 0, 10 or 20 μg/ml proteinase K on ice. After 10 min PMSF was added to samples to a final concentration of 2.5 mM and incubated for further 10 min. Mitochondria were washed with SEM buffer and resuspended in Laemmli buffer (120 mM Tris-HCl, 20% (v/v) glycerol, 4% (w/v) SDS, 0.001% (w/v) bromophenol blue; 3% (v/v) β-mercaptoethanol; pH 6.8). Samples were analyzed by SDS-PAGE and immunoblotting, according to standard protocols.

### Statistical Analysis

To analyze the effect of dependent variables across the type of expression (between-subject factor) and along time (within-subject factor), a two-way mixed-design analysis of variance (ANOVA) with a Bonferroni *post hoc* test was applied. To disprove the null-hypothesis (no difference between conditions) in non-time-depending experiments, a one-way ANOVA corrected with a Bonferroni *post hoc* test for one variable (type of expression) was performed. A Student’s *t*-test was applied to compare between two groups. Significances are indicated with asterisks: ***p < 0.001, **p < 0.01, *p < 0.05, not significant (n.s.) *p* > 0.05. Final results of experiments, performed at multiple time points, are visualized as fold-values normalized to control cells at the earliest time point shown. Statistical analysis was performed with Origin Pro 2016 (OriginLab) and figures were prepared with Origin Pro 2016 and Adobe Illustrator CS6 (Adobe).

## Results

### Cellular Age Is a Decisive Factor for LRRK2^RCK^-Induced Death

Within the last 15 years, numerous humanized yeast models for neurodegeneration have been developed, not only successfully recapitulating basic characteristics of diverse neurotoxic proteins, but also providing new insights into the molecular pathways and determinants underlying disease pathology that subsequently were found to be conserved across species (Braun et al., 2010; Oliveira et al., 2017; Tenreiro et al., 2017). While the heterologous expression of the enzymatic core of LRRK2 in dividing yeast cells has been shown to cause growth arrest and defects in vesicular trafficking (Xiong et al., 2010), nothing is known about its effect on post-mitotic yeast cells. Thus, we established a setup that allowed us to analyze LRRK2-mediated changes in stationary, aging cells. Here, the galactose-driven expression of a truncated version of LRRK2, ranging from amino acid 1300–2163 and containing its ROC, COR and kinase domain (LRRK2^RCK^, Figure 1A) did not affect exponential growth (Figure 1B). Instead, upon entry into stationary phase, LRRK2^RCK^ triggered a progressive loss of survival starting at day 3 as determined by flow cytometric quantification of membrane rupture (Figure 1C) and clonogenic assays monitoring the abundance of viable cells (Figure 1D). The point mutation R1398L partly prevented, but not completely inhibited LRRK2^RCK^ cytotoxicity. This mutation has been shown to convey higher GTPase activity (Xiong et al., 2010), and an exchange at the R1398 residue has been identified as the functional variant in a human haplotype that is protective against PD (Ross et al., 2011; Heckman et al., 2014). Expression of a protein of comparable size (ß-galactosidase, LacZ) did not affect survival during aging compared to the empty vector control (Figure 1C) and was used as control for further experiments. The pathogenic point mutation G2019S, which has been shown to increase kinase activity (West et al., 2005), seemed to slightly enforce LRRK2^RCK^-mediated cell death, however without reaching statistical significance (Supplementary Figures S1A–G).

To discriminate between necrotic and early/late apoptotic cell death, we performed AnnexinV/PI co-staining, demonstrating that LRRK2^RCK^-mediated age-dependent death displays phenotypic manifestation of both apoptosis and necrosis (Figures 1E,F and Supplementary Figures S1D–F). Conclusively, the R1398L^RCK^ mutant displayed reduced cytotoxicity, which was not due to decreased stability or expression of this variant as shown by immunoblots decorated with an antibody against the C-terminal V5 tag of the proteins (Figure 1G). In addition, these experiments revealed the presence of dimeric and trimeric species of LRRK2^RCK^ variants already on day 1 (Figure 1G and Supplementary Figure S1G).

Thus, LRRK2^RCK^ triggers age-dependent cell death that can be partly prevented by a mutation conferring increased GTPase activity and, comparable to full-length LRRK2 in mammalian cells (Islam and Moore, 2017), dimerizes in yeast.

### LRRK2^RCK^ Localizes to Mitochondria and the Plasma Membrane

A broad cellular localization has been reported for LRRK2, ranging from microtubules, membranes and diverse vesicular structures to mitochondria (Biskup et al., 2006; Berger et al., 2010; Ramírez et al., 2017). We analyzed the localization of C-terminally mCherry-tagged LRRK2^RCK^ and R1398L^RCK^ in yeast strains expressing endogenously GFP-tagged marker proteins for different organelles with three-dimensional confocal laser scanning microscopy. In accordance with its localization in higher eukaryotic cells, we find a portion of LRRK2^RCK^ (and of the R1398L^RCK^ variant) associated with the plasma membrane, as revealed by simultaneous visualization of GFP-labeled Pma1, the major plasma membrane H^+^-ATPase (Figure 2A). However, the majority of LRRK2^RCK^ accumulated at intracellular structures that neither co-localized with vacuoles, visible due to the transport of Pma1-GFP to the vacuole for subsequent degradation (Chang and Fink, 1995; Henderson et al., 2014; Figure 2A), nor the nucleus, visualized by a histone-GFP chimera (Supplementary Figure S2A). Instead, these structures co-localized with Om45, a protein of the outer mitochondrial membrane (Figure 2B). Quantitative analysis of microscopic pictures confirmed that the majority of the LRRK2^RCK^-mCherry signal overlapped with the Om45-GFP signal (M2 = 0.74) and, to a lower extend, also with Pma1-GFP (M2 = 0.17, please see Supplementary Figure S2B for detailed quantification). Of note, Pma1 exhibited a raft-like distribution at the plasma membrane as shown in Figure 2A and reported previously (Spira et al., 2012). While also LRRK2^RCK^ localized in distinct clusters at the plasma membrane (Figure 2A), Pma1 and LRRK2^RCK^ spots appeared rather separated and the quantification of Pma1 and LRRK2^RCK^ colocalization might thus underestimate the actual portion of this protein at the plasma membrane. In line with studies reporting severe impairment of mitochondrial morphology upon expression of full-length LRRK2 or its pathogenic variants (Ramonet et al., 2011; Su and Qi, 2013), the yeast mitochondrial network appeared fragmented and aggregated upon LRRK2^RCK^ expression (Figure 2B). Next, we subjected cells expressing V5-tagged LRRK2^RCK^ to subcellular fractionation. LRRK2^RCK^ showed a broad distribution, and the most prominent bands were detectable in mitochondrial and plasma membrane fractions, confirming the microscopy results (Figure 2C). Furthermore, we could detect LRRK2^RCK^ in isolated mitochondria (Figure 2D). Treatment of these mitochondrial fractions with proteinase K demonstrated that LRRK2^RCK^, similar to the intermembrane space (IMS) control protein Mcr1, was not accessible to the protease, indicating that LRRK2^RCK^ resides within mitochondria. In sum, these data suggest an association of LRRK2^RCK^ with the plasma membrane and mitochondria.

### Mitochondrial Function Is Impaired Upon LRRK2^RCK^ Expression

We further analyzed mitochondrial functionality in respect to respiration, generation of ROS and transmembrane potential. Measurement of cellular oxygen consumption via a Fire-Sting optical oxygen sensor system revealed a drop of respiration caused by LRRK2^RCK^ on day 2. While control cells displayed a massive increase in oxygen consumption with progressing age, reflecting the induction of the aerobic metabolism after the diauxic shift, this boost in respiration was reduced in cells expressing LRRK2^RCK^ (Figure 3A). The ROS-driven conversion of non-fluorescent dihydroethidium (DHE) to fluorescent ethidium (Eth) demonstrated that enhanced oxygen consumption of control cells was accompanied by an increase in oxidative stress (Figure 3B). While LRRK2^RCK^ expressing cells exhibited lower respiratory activity, the production of ROS was still enhanced compared to control cells on day 2, suggesting a dysfunction in oxidative phosphorylation. Dead cells, which randomly accumulate the dye, were excluded from the analysis as depicted in Figure 3C. Quantification of total cellular ATP content revealed reduced ATP levels upon prolonged expression of LRRK2^RCK^ (Figure 3D). The R1398L point mutation mitigated the LRRK2^RCK^-driven effects on respiration, oxidative stress and cellular ATP levels (Figures 3A–D). Moreover, mitochondrial transmembrane potential (ΔΨ_m_) assessed by microscopy and flow cytometry on day 2 of aging was reduced upon LRRK2^RCK^ expression, whereas these changes were less pronounced in R1398L^RCK^ expressing cells (Figures 3E,F).

To further elucidate the relation between mitochondrial dysfunction and cell death caused by LRRK2^RCK^, we compared the so-far used BY4741 strain to the more respiratory active strain W303. In the W303 background, LRRK2^RCK^ triggered cell death already on day 1, despite comparable LRRK2^RCK^ expression levels in BY4741 and W303 strains (Figures 3G,H). Respiration was increased 30-fold in control cells of the W303 strain compared to BY4741 control cells (Figure 3I), suggesting that cells with high respiratory activity are particularly sensitive to LRRK2^RCK^. Again, the R1398L^RCK^ variant displayed reduced cytotoxicity. In sum, LRRK2^RCK^ disrupts mitochondrial respiration, leading to oxidative stress and energy depletion.

### LRRK2^RCK^ Reduces Mitochondrial Abundance

**Figure 4:**
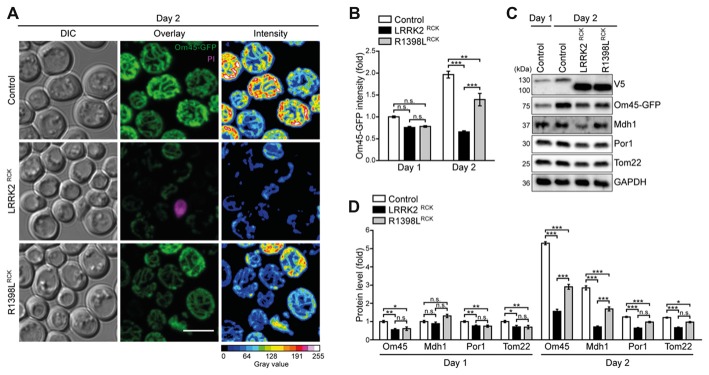
Mitochondrial morphology and abundance is impaired by LRRK2^RCK^. **(A,B)** Analysis of cells harboring endogenously GFP-tagged Om45 expressing LacZ, LRRK2^RCK^ or R1398L^RCK^. Representative confocal micrographs on day 2 **(A)** and flow cytometric quantification of Om45-GFP fluorescence signal at day 1 and 2 **(B)** are shown. Dead cells were excluded via PI counterstaining. Values were normalized to control cells on day 1. Scale bar represents 5 μm. Means ± SEM; *n* = 4. For confocal micrographs of Om45-GFP strains on day 1, please see Supplementary Figure S3A. **(C,D)** Immunoblot analysis of extracts from cells as described in **(A)**. Representative immunoblots **(C)** and densitometric quantification **(D)** are shown. Blots were probed with antibodies directed against the V5- and the GFP-epitopes, against the mitochondrial proteins Mdh1, Por1 and Tom22, and against glyceraldehyde 3-phosphate dehydrogenase (GAPDH) as loading control. Values were normalized to the respective signals from control cells on day 1. Means ± SEM; *n* ≥ 6. For immunoblot analysis of Om45-GFP cells on day 1, and of Tim44-GFP strains, please see Supplementary Figures S3B–E. ***p < 0.001, **p < 0.01, *p < 0.05, n.s. not significant.

The decrease in respiratory capacity and cellular ATP levels prompted us to analyze mitochondrial morphology and abundance at early time points during aging using microscopy and flow cytometry. While only a mild impairment of the mitochondrial appearance was visible after entry into stationary phase (Supplementary Figure S3A), we observed a severely aggregated and fragmented mitochondrial network on day 2 of LRRK2^RCK^ expression, thus preceding the onset of LRRK2^RCK^-induced cell death (Figure 4A). These effects were again less prominent in cells expressing R1398L^RCK^. Consistently, flow cytometric quantification of Om45-GFP fluorescence intensity indicated a prominent reduction of mitochondrial mass starting on day 2 (Figure 4B). Control cells increased their Om45-GFP levels between day 1 and day 2, reflecting the augmentation of mitochondrial mass after the diauxic shift, when cells rely on respiration for energy production. This increase was absent in cells expressing LRRK2^RCK^. Similar results were obtained using endogenously GFP-tagged Tim44, a component of the translocase of the inner mitochondrial membrane, as readout (Supplementary Figure S3B). Immunoblot analysis was used to determine the protein levels of Om45-GFP and Tim44-GFP and confirmed these results. The reduction of several additional mitochondrial proteins demonstrated a general decrease of mitochondrial abundance induced by LRRK2^RCK^, again less prominent for the R1398L^RCK^ mutant (Figures 4C,D, for immunoblots on day 1 and quantification of Tim44-GFP levels, please see Supplementary Figures S3C–E), while the pathogenic G2019S^RCK^ variant impaired mitochondrial abundance to a similar extend than the wild type LRRK2^RCK^ (Supplementary Figures S4A,B).

The observed depletion of mitochondria might underlie the decrease in respiration and ATP levels, and our data points towards inefficient replenishment of the mitochondrial population when cells need to adapt their metabolism to respiration.

### LRRK2^RCK^ Inhibits Mitochondrial Biogenesis

**Figure 5:**
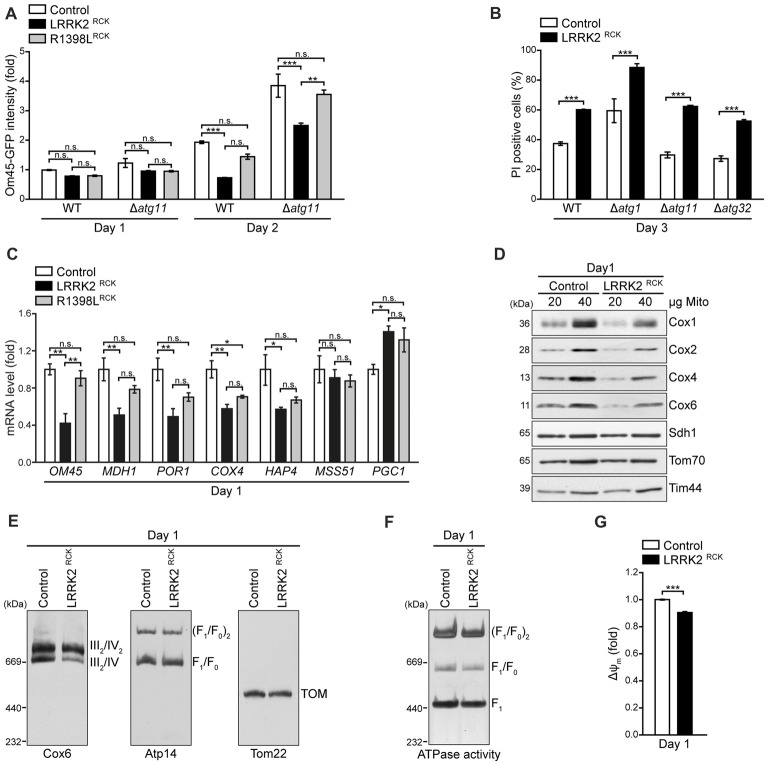
LRRK2^RCK^ compromises mitochondrial biogenesis and complex IV assembly. **(A)** Flow cytometric quantification of wild type and *ATG11* deletion strains harboring Om45-GFP and expressing LacZ, LRRK2^RCK^ or R1398L^RCK^ on day 1 and 2 of aging. Dead cells were excluded via PI counterstaining. Values were normalized to control cells on day 1. Means ± SEM; *n* = 4. **(B)** Flow cytometric quantification of PI-stained wild type and *ATG1*, *ATG11* and *ATG32* deletion strains expressing LacZ or LRRK2^RCK^ on day 3 of aging. Means ± SEM; *n* = 4. **(C)** q-RT-PCR to determine mRNA levels of indicated mitochondria-related transcripts in cells expressing LacZ, LRRK2^RCK^ or R1398L^RCK^. Normalization was performed using mRNA levels of *UBC6*. Means ± SEM; *n* = 4. **(D)** Immunoblot analysis of mitochondria isolated from cells expressing LacZ or LRRK2^RCK^ on day 1. Blots were probed with antibodies directed against the respiratory chain complex IV components Cox1, Cox2, Cox4 and Cox6, as well as Sdh1 (subunit of complex II), Tom70 (outer mitochondrial membrane) and Tim44 (inner mitochondrial membrane). **(E)** Immunoblots of Blue-Native-PAGE with samples described in **(D)**. Blots were probed with antibodies against Cox6 (complex III/IV), Atp14 (complex V) and Tom22 (translocase of outer membrane (TOM) complex). **(F)** In-gel ATPase activity assay of samples described in **(D)**. **(G)** Measurement of mitochondrial transmembrane potential (ΔΨ_m_) in mitochondria isolated from cells described in **(D)**. Values for ΔΨ_m_ were normalized to mitochondria from control cells. Means ± SEM; *n* = 3. ***p < 0.001, **p < 0.01, *p < 0.05, n.s. not significant.

Reduced mitochondrial abundance upon LRRK2^RCK^ expression might be due to excessive mitochondrial degradation or to insufficient mitochondrial biosynthesis. To evaluate the contribution of increased mitochondrial degradation via selective autophagy (mitophagy) to mitochondrial depletion, we monitored Om45-GFP intensity as a readout for mitochondrial mass in strains lacking Atg11, an essential adapter protein for selective autophagic processes, including mitophagy. While the absence of Atg11 amplified mitochondrial mass in general, it did not affect the LRRK2^RCK^-driven reduction of Om45-GFP signal, indicating that the loss of mitochondrial mass is not due to mitochondrial degradation via mitophagy (Figure 5A). Examining cytotoxicity in cells devoid of Atg11 as well as in strains lacking Atg32, a mitochondrial outer membrane (OM) protein crucial for the initiation of mitophagy, or Atg1, an essential component of the general autophagic machinery, demonstrated that LRRK2^RCK^-mediated death of aging cells was unaltered in these mutants (Figure 5B). Next, we determined the levels of several mitochondria-related transcripts upon expression of LRRK2^RCK^. This revealed a transcriptional downregulation of a diverse set of mitochondrial proteins, including *OM45*, the mitochondrial malate dehydrogenase *MDH1*, the porin *POR1*, and a subunit of respiratory chain complex IV,* COX4* (Figure 5C). The levels of *HAP4*, an important transcription factor required for reprogramming yeast cells from fermentation to respiration (Buschlen et al., 2003), were also reduced. Interestingly, we found LRRK2^RCK^ to upregulate mRNA levels for *PGC1* encoding the phosphatidylglycerol phospholipase C that regulates mitochondrial phosphatidylglycerol content to ensure proper mitochondrial function and morphology (Simocková et al., 2008; Pokorná et al., 2016). Thus, LRRK2^RCK^ does not affect mitophagic degradation but globally impairs mitochondrial biogenesis and this can, at least in part, be explained by changed transcription.

### LRRK2^RCK^ Causes Misassembly of the Respiratory Chain Complex IV

While the reduced respiration as well as the drop of ATP levels most probably arise from a general decrease in mitochondrial mass, the LRRK2^RCK^-driven accumulation of ROS (Figure 3B) points to additional defects in oxidative phosphorylation in the residual mitochondrial population. Therefore, we purified mitochondria and analyzed the levels of mitochondrial proteins early after entry into the post-mitotic state (day 1). While the levels of Sdh1, a subunit of the respiratory chain complex II, and of Tom70 and Tim44 as outer and inner mitochondrial membrane proteins, respectively, were unchanged, we observed a reduction of all tested components of the respiratory chain complex IV (cytochrome c oxidase) upon LRRK2^RCK^ expression (Figure 5D). Moreover, Blue-Native PAGE demonstrated that LRRK2^RCK^ selectively impaired the assembly of complex IV. In contrast, the translocase of the outer membrane (TOM complex) as well as assembly and ATPase activity of complex V (mitochondrial F_1_F_0_ ATP synthase) were not affected by LRRK2^RCK^ (Figures 5E,F). Notably, while the outer mitochondrial membrane was intact (Figure 2D), mitochondria purified from cells expressing LRRK2^RCK^ displayed a slight dissipation of ΔΨ_m_ compared to mitochondria from control cells (Figure 5G), most likely a result of impaired complex IV assembly. Thus, already early during aging, LRRK2^RCK^ selectively compromises complex IV formation, with subsequent consequences for mitochondrial function and generation of ROS.

## Discussion

PD-associated neurotoxic consequences of LRRK2 are thought to be due to a gain-of-function of the protein, emanating from pathogenic mutations (e.g., G2019S with increased kinase activity) or from elevated protein loads, particularly found in brains of patients in early stages of PD (Dzamko et al., 2017). Mitochondrial dysfunction and reduced mitochondrial mass have been frequently reported upon dysregulation of LRRK2 and have mostly been attributed to excessive mitochondrial degradation (Cherra et al., 2013). Using aging yeast cells, we show that LRRK2^RCK^ diminishes mitochondrial biogenesis and provide insights into the progressive decline of mitochondrial function as a consequence of cellular age. While heterologous expression of LRRK2^RCK^ in proliferating yeast has been shown to arrest growth (Xiong et al., 2010), we established a model in which LRRK2^RCK^ does not affect proliferation but impacts cellular fitness in stationary phase, recapitulating the situation in post-mitotic cells such as neurons (Carmona-Gutierrez and Büttner, 2014).

**Figure 6:**
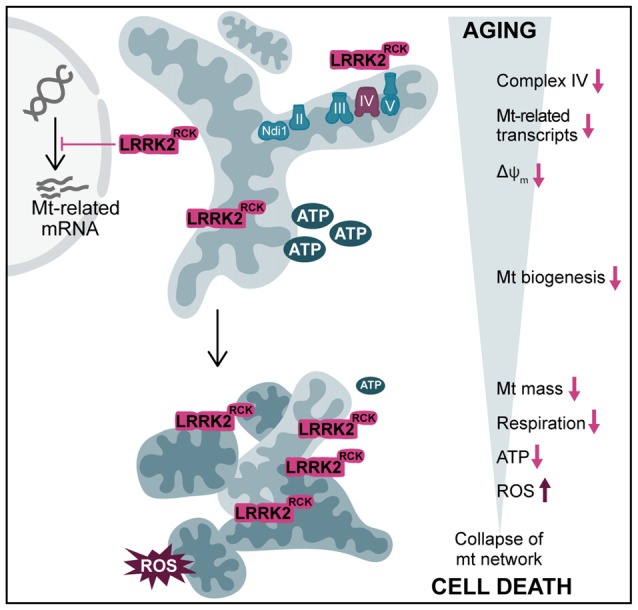
Overview of LRRK2^RCK^-induced mitochondrial dysfunction in yeast. In post-mitotic yeast cells, LRRK2^RCK^ (magenta) is targeted to mitochondria, leading to misassembly of respiratory chain complex IV (cytochrome c oxidase). Transcription of mitochondria (mt)-related gene products is reduced and a dissipation of the mitochondrial transmembrane potential (ΔΨ_m_) can be observed. With increasing age, cells display a prominent decrease in mt mass and a concomitant drop in respiratory activity and ATP levels. In addition, mitochondrial dysfunction leads to enhanced production of ROS. This culminates in the collapse of the mt network, ultimately resulting in cell death.

Based on our findings, we propose the following sequence of events resulting in death of aging cells: LRRK2^RCK^ is targeted to mitochondria and causes a specific decrease in the levels of complex IV components. In consequence, complex IV is insufficiently assembled and ΔΨ_m_ starts to dissipate, while the ATPase activity of complex V remains unaffected. Moreover, a transcriptional downregulation of diverse mitochondrial gene products becomes evident. This happens shortly after entry into a post-mitotic state, where cells induce the amplification of mitochondrial mass to adjust to the need for respiration as energy source. With progressing age, these early defects culminate in: (i) a reduction of mitochondrial mass, (ii) insufficient respiratory capacity, (iii) a further drop in ΔΨ_m_, (iv) mitochondrial fragmentation, (v) decreased cellular ATP content, and (vi) oxidative stress. Ultimately, these LRRK2^RCK^-mediated changes lead to cell death (Figure 6). While our results clearly link LRRK2^RCK^-induced cellular demise to defects in complex IV and a reduction of mitochondrial biogenesis, further research is needed to establish the precise sequence of events. The complex IV deficiency might very well represent an early manifestation of the decrease in mitochondria-related gene transcripts, including *COX4*. Vice versa, it is also feasible that mitochondria-resident LRRK2^RCK^ directly targets complex IV and that this in turn activates mitochondria-to-nucleus signaling, thereby altering transcription.

So far, rather pleiotropic effects of LRRK2 on mitochondria were observed, ranging from no alteration of mitochondrial content but dissipation of ΔΨ_m_ (Mortiboys et al., 2010; Papkovskaia et al., 2012) to a reduction of mitochondrial mass within neurites via either increased retrograde trafficking (Schwab et al., 2017), decreased anterograde trafficking (Godena et al., 2014; Thomas et al., 2016), or excessive degradation of mitochondria via mitophagy (Cherra et al., 2013). Although, enforcing mitochondrial biogenesis via activation of the PGC-1α ortholog in *Drosophila melanogaster* resulted in a reduction of PD phenotypes induced by Parkin and LRRK2 (Ng et al., 2017), LRRK2-mediated effects on mitochondrial biogenesis remained unexplored. In our experiments, genetic inactivation of mitophagy did neither prevent the loss of mitochondrial mass nor cytotoxicity driven by LRRK2^RCK^. Instead, we find a downregulation of various mitochondrial transcripts, including *HAP4*, the transcriptional activator of global respiratory gene expression. In consequence, this entails inefficient amplification of mitochondrial mass to increase respiratory capacity in stationary phase. These data establish inhibition of mitochondrial biogenesis rather than enhanced mitochondrial degradation as primary cause for the drop in mitochondrial abundance upon LRRK2^RCK^ expression.

Several studies show that LRRK2 influences transcription (Dorval and Hébert, 2012), but the mechanistic details of these changes and their contribution to PD pathology remain elusive. Interestingly, LRRK2-mediated transcriptional alterations are also associated with Crohn’s disease, a type of inflammatory bowel disease (Liu et al., 2011), and LRRK2 alleles contributing to both PD and Crohn’s disease have been identified (Hui et al., 2018). Mice lacking LRRK2 display enhanced nuclear translocation of the nuclear factor of activated T cells (NFAT), suggesting that a physiological function of LRRK2 is to regulate the cellular distribution and thus activity of NFAT and that loss of this regulation contributes to the progression of Crohn’s disease (Liu et al., 2011). In addition, genome-wide mRNA profiling identified transcriptional changes for both LRRK2 deficiency and pathogenic mutation, showing differential gene expression profiles for a wide range of cellular processes, including ribosomal/translational function, glycolysis, ubiquitination and oxidative phosphorylation (Nikonova et al., 2012). Thus, it is feasible that LRRK2 modulates the nuclear translocation or activity of distinct transcription factors, such as regulators of *HAP4*, resulting in decreased mitochondrial biogenesis. On the other hand, early and subtle mitochondrial damage by LRRK2 might interfere with the expression of mitochondria-encoded proteins, culminating in the observed complex IV misassembly which in consequence might also trigger a compensatory downregulation of nuclear-encoded mitochondria-related transcripts.

Whereas in proliferating yeast cells a cytosolic localization of LRRK2^RCK^ has been reported (Xiong et al., 2010), we find LRRK2^RCK^ and the R1398L^RCK^ variant associated with mitochondria and the plasma membrane in post-mitotic cells, recapitulating the localization of full-length LRRK2 in higher eukaryotic systems (Biskup et al., 2006; Berger et al., 2010; Ramírez et al., 2017). While proteinase K treatment of isolated mitochondria suggests that LRRK2^RCK^ resides within these organelles, this might also reflect proteinase K-resistant LRRK2^RCK^ aggregates attached to the outer mitochondrial membrane. Further analyses are needed to determine the exact localization of distinct LRRK2^RCK^ species in or at mitochondria and how this relates to decreased complex IV abundance and assembly. Interestingly, mRNA levels of the *PGC1* gene, which encodes a phosphatidylglycerol phospholipase, were upregulated in response to LRRK2^RCK^ expression. Pgc1 is important to maintain balanced ratios of anionic phospholipids ensuring proper mitochondrial function and morphology, and deletion of *PGC1* has been shown to increase complex IV activity (Pokorná et al., 2016). Thus, the LRRK2^RCK^-mediated upregulation of *PGC1* might contribute to the misassembly of complex IV in aging yeast.

Notably, while the G2019S^RCK^ variant triggered cell death comparable to the wild type form of LRRK2^RCK^, the R1398L^RCK^ point mutant displayed decreased cytotoxicity, indicating a cytoprotective role of high GTPase activity (Xiong et al., 2010; Biosa et al., 2013; Nguyen and Moore, 2017). Though genetic variations at the R1398 site have been shown to decrease the risk to develop PD (Ross et al., 2011; Heckman et al., 2014), the differential contribution of the two enzymatic activities of LRRK2 to the neurodegenerative processes during PD is not fully understood and might be rather complex. GTP-binding as well as GTP-hydrolysis of the ROC domain has been shown to regulate kinase activity, and vice versa, the kinase of LRRK2 autophosphorylates distinct sites in the ROC domain, thereby driving GTP-hydrolysis (Biosa et al., 2013). In addition, this intricate regulation of the two catalytic functions is likely modulated by distinct effector proteins and the specific cellular context (Liu et al., 2018). Interestingly, LRRK2 toxicity in neurons depends on the presence of α-synuclein (Skibinski et al., 2014), and vice versa rats devoid of LRRK2 are more vulnerable to dopaminergic neurodegeneration caused by high levels of α-synuclein (Daher et al., 2014), providing evidence for an interplay between these two PD-associated proteins and a function of LRRK2 that can counteract α-synuclein-associated neurodegeneration. As α-synuclein also has been shown to compromise mitochondrial function, the modulation of mitochondrial biogenesis via LRRK2 might thus impact distinct aspects of α-synuclein-driven cellular demise.

In sum, we show that inhibition of mitochondrial biogenesis rather than increased mitochondrial degradation is causative for the LRRK2^RCK^-mediated decline in mitochondrial mass. With progressing cellular age, LRRK2^RCK^ triggers a sequence of events that includes distinct transcriptional alterations and a drop in complex IV abundance, followed by a decrease in mitochondrial transmembrane potential and respiration, accumulation of ROS and a breakdown of the mitochondrial network, ultimately resulting in cell death.

## Author Contributions

AA, VK and SB conceptualized the study and analyzed the data and wrote the manuscript. AA, VK, CW, ST-C and LH performed the experiments. F-NV and WK analyzed and discussed the data and gave conceptual advice. SB supervised the study. All authors commented on the manuscript, read and approved the final version.

## Conflict of Interest Statement

The authors declare that the research was conducted in the absence of any commercial or financial relationships that could be construed as a potential conflict of interest.

## Abbreviations

ΔΨ_m_: mitochondrial transmembrane potential
COR: C-terminal-of-ROC
DHE: dihydroethidium
Eth: ethidium
GFP: green fluorescent protein
IMS: intermembrane space
LRRK2: leucine-rich repeat kinase 2
OM: outer (mitochondrial) membrane
PD: Parkinson’s disease
PI: propidium iodide
ProtK: proteinase K
ROC: Ras-of-complex
ROS: reactive oxygen species.
