# Supplementary material for: The Enzymatic Core of the Parkinson’s Disease-Associated Protein LRRK2 Impairs Mitochondrial Biogenesis in Aging Yeast
Source: Front Mol Neurosci. 2018 Jun 21;11:205. doi: 10.3389/fnmol.2018.00205 (PMC6021522; doi:10.3389/fnmol.2018.00205)
Supplement: Supplementary file 1 [file Data_Sheet_1.pdf]

Supplementary Material

The Enzymatic Core of the Parkinson's Disease-Associated Protein LRRK2 Impairs Mitochondrial Biogenesis in Aging Yeast

Andreas Aufschnaiter[1,#], Verena Kohler[1,#], Corvin Walter[2,3], Sergi Tosal-Castano[4], Lukas Habernig[4], Heimo Wolinski[1], Walter Keller[1], F.-Nora Vögtle[2] and Sabrina Büttner[1,4,*]

[1] Institute of Molecular Biosciences, University of Graz, Graz, Austria

[2] Institute of Biochemistry and Molecular Biology, ZBMZ, Faculty of Medicine, University of Freiburg, Germany

[3] Faculty of Biology, University of Freiburg, Germany

[4] Department of Molecular Biosciences, The Wenner-Gren Institute, Stockholm University, Stockholm, Sweden

[#] These authors contributed equally to this work.

[*] Correspondence:
Sabrina Büttner
sabrina.buettner@su.se

Contains:

Supplementary Figures S1-S4
Supplementary Tables S1 and S2

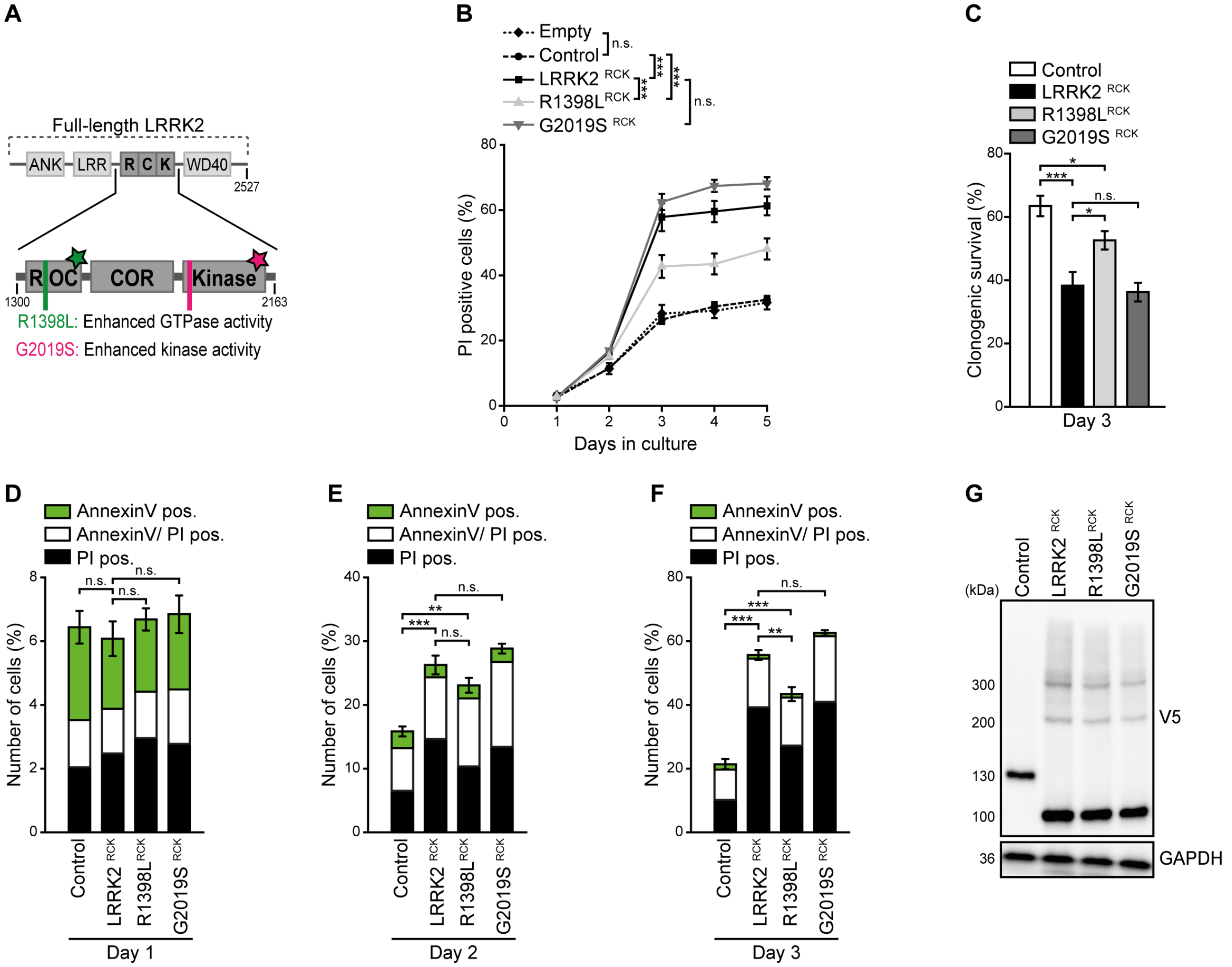

Supplementary Figure S1: The G2019S point mutation does not affect age-dependent cell death caused by LRRK2^{RCK}.

(A) Scheme of truncated LRRK2 constructs used in this study. The enzymatic core of human LRRK2 (amino acids 1300 to 2163) containing the ROC (Ras-of-complex) GTPase, the COR (C-terminal-of-ROC) and the protein kinase domain (together LRRK2^{RCK}), was expressed in yeast cells. Wild type as well as the mutant forms R1398L^{RCK} with higher GTPase activity and G2019S^{RCK}, conveying enhanced kinase activity, were used. The green star indicates GTPase-, the star in magenta represents kinase activity.

(B) Flow cytometric quantification of propidium iodide (PI) stained cells expressing either LacZ as a control or LRRK2^{RCK} and its variants described in **(A)**. Cells harboring the empty vector were analyzed to validate the suitability of LacZ expression as a control. Significances represent simple main effects between different expression types at each time point. Significances shown are valid for day 3-5. Means ± SEM; n=4.

(C) Clonogenic survival on day 3 of cells described in **(B)** Means ± SEM; n=6.

(D-F) Flow cytometric quantification of AnnexinV/PI co-staining at indicated time points during chronological aging. Means ± SEM; n=4.

(G) Immunoblot analysis of protein extracts from cells described in **(B)**. Blots were probed with antibodies directed against the V5-epitope to detect V5-tagged LacZ and LRRK2^{RCK} variants and against glyceraldehyde 3-phosphate dehydrogenase (GAPDH) as a loading control.

*** $p<0.001$, ** $p<0.01$, * $p<0.05$, n.s. not significant.

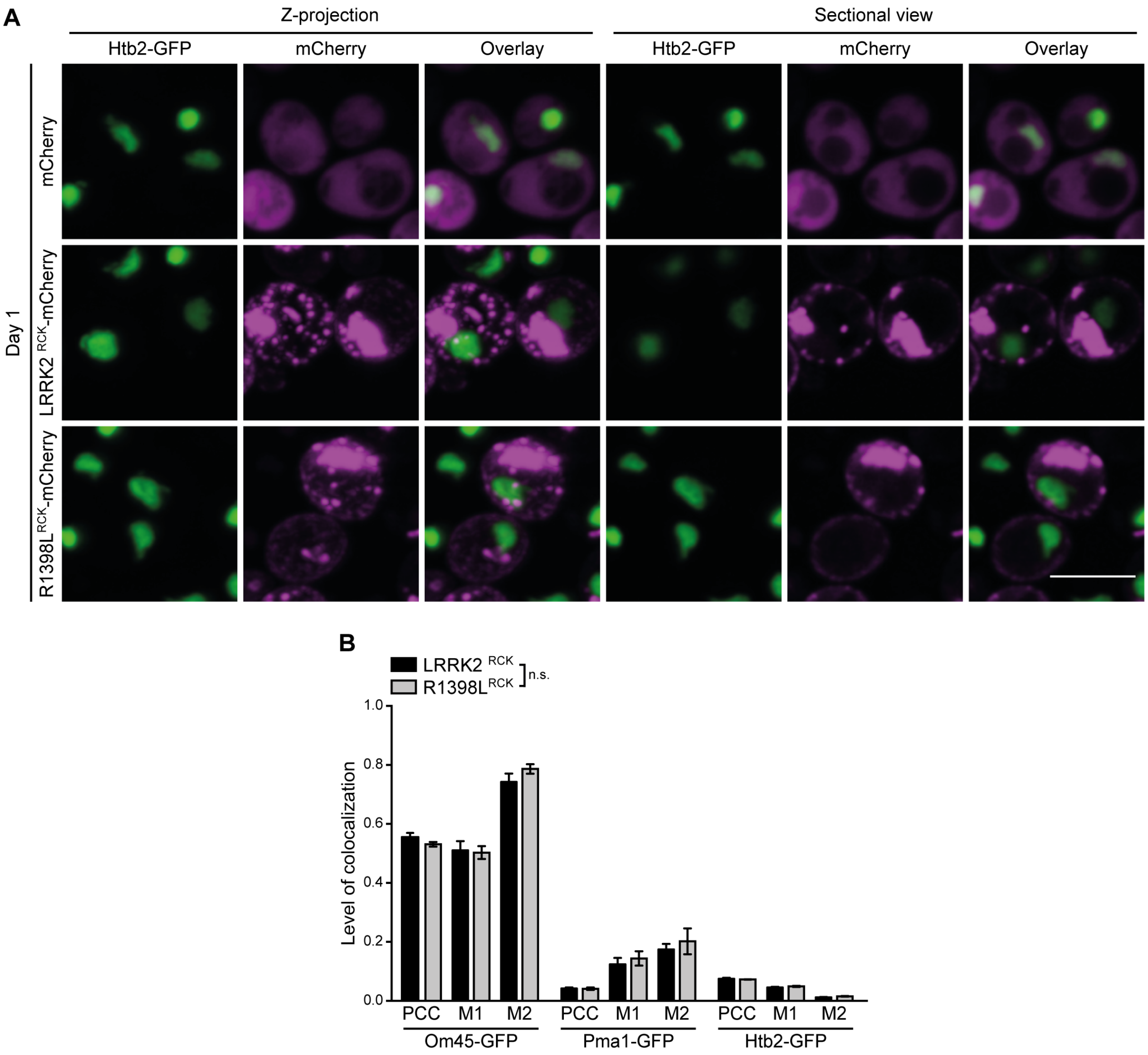

Supplementary Figure S2: LRRK2^{RCK} does not localize in the nucleus.
(A) Representative confocal micrographs of strains harboring endogenously GFP-tagged Htb2 and expressing mCherry alone or fused to LRRK2^{RCK} and R1398L^{RCK} on day 1 of aging. Z-projections of three-dimensional stacks are shown, as well as a representative section. Scale bar represents 5 µm.
(B) Quantification of colocalization from microscopic pictures of cells expressing mCherry fused to LRRK2^{RCK} and R1398L^{RCK} on day 1 of aging, harboring endogenously GFP-tagged Om45 (representative pictures in Figure 2B), Pma1 (Figure 2A) or Htb2 (Supplementary Figure S2A). Pearson correlation coefficient (PCC) as well as Manders' coefficient M1 (overlap of GFP signal with LRRK2^{RCK}- or R1398L^{RCK}-mCherry) and M2 (overlap of LRRK2^{RCK}- or R1398L^{RCK}-mCherry with GFP signal) are shown. For each strain and expression type, at least 120 cells from three different clones were analyzed. Means ± SEM; *n*=3; n.s. not significant.

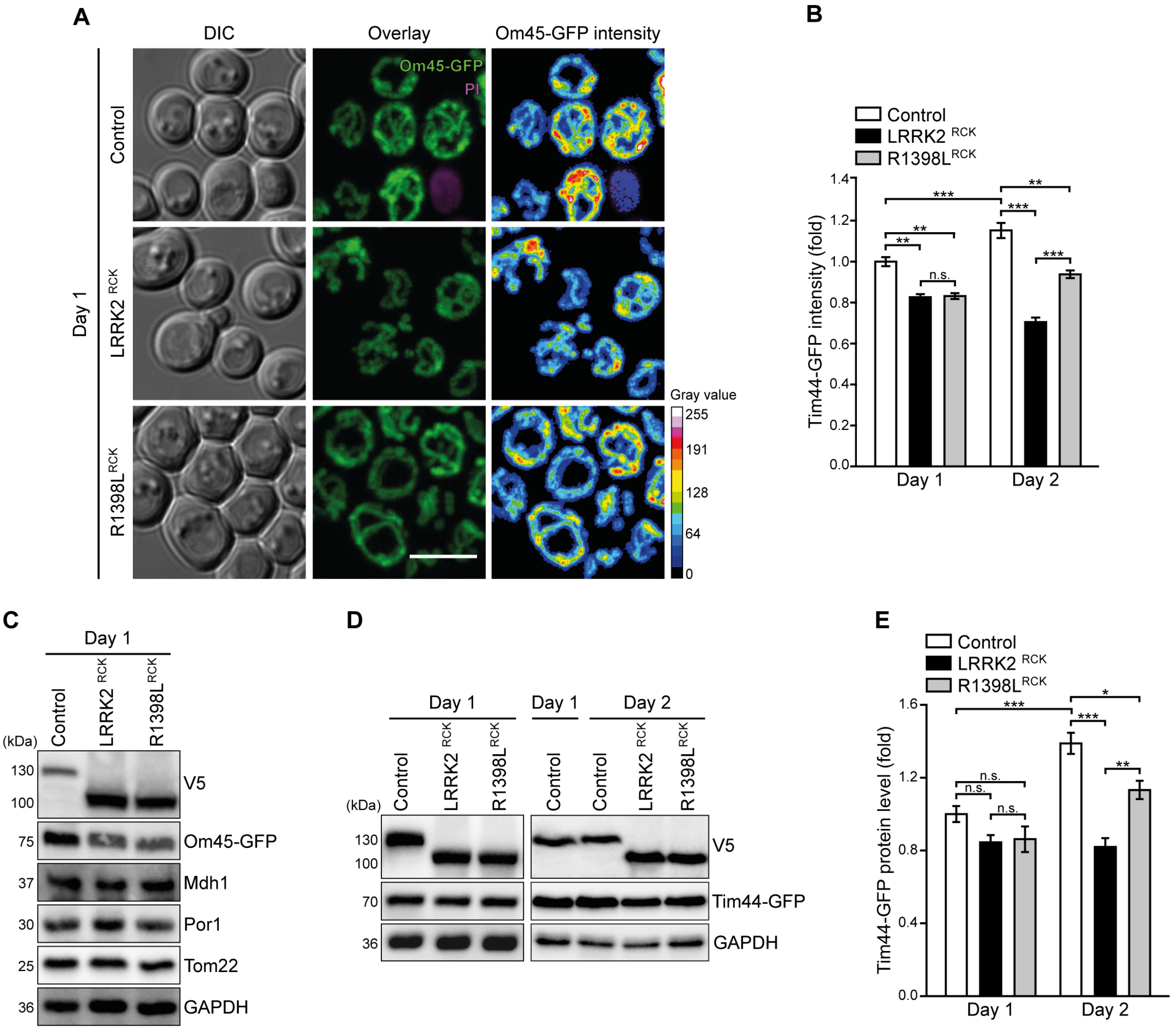

Supplementary Figure S3: LRRK2^{RCK} alters mitochondrial morphology and abundance.
(A) Microscopic analysis of fluorescence signal in strains harboring endogenously C-terminally GFP-tagged Om45, expressing LacZ, LRRK2^{RCK} or R1398L^{RCK}. Representative confocal micrographs of day 1 are shown. Dead cells were visualized via propidium iodide (PI) counterstaining. Scale bar represents 5 µm.
(B) Flow cytometric quantification of the mean fluorescence intensity of cells harboring endogenously C-terminally GFP-tagged Tim44, expressing LacZ, LRRK2^{RCK} or R1398L^{RCK}. Intensities were normalized to control cells on day 1. Dead cells were excluded from the analysis via PI counterstaining. Means ± SEM; *n*=4.
(C) Immunoblot analysis of cells as described in **(A)**. Blots were probed with antibodies against the V5- and the GFP-epitope, against the mitochondrial proteins Mdh1, Por1 and Tom22, and against glyceraldehyde 3-phosphate dehydrogenase (GAPDH) as loading control.
(D, E) Immunoblot analysis of cells as described in **(B)**. A representative immunoblot **(D)** and densitometric quantification **(E)** are shown. Blots were probed with antibodies against the GFP-epitope and against GAPDH as loading control. Values were normalized to the average of the respective signals from control cells on day 1. Means ± SEM; $n \geq 10$.
*** $p<0.001$, ** $p<0.01$, * $p<0.05$, n.s. not significant.

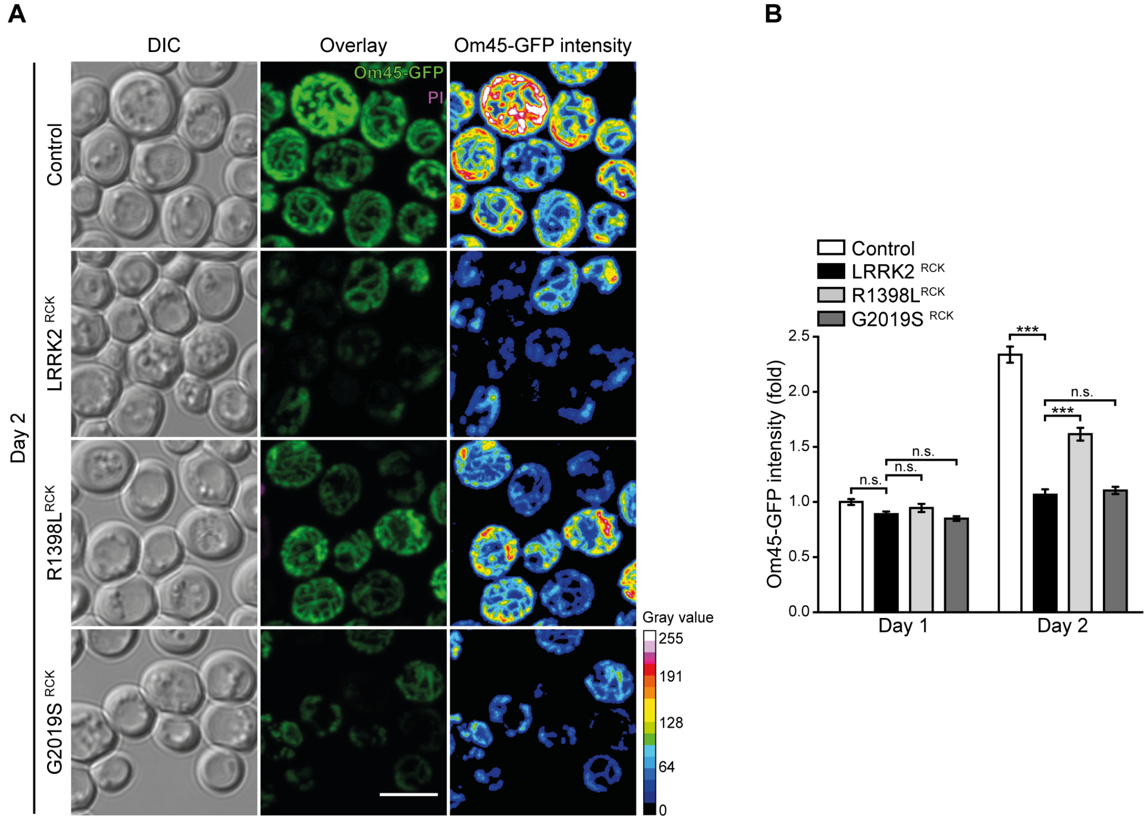

Supplementary Figure S4: The G2019S mutation does not affect LRRK2^{RCK}-mediated alterations in mitochondrial morphology and abundance.

(A) Microscopic analysis of fluorescence signal in strains harboring endogenously C-terminally GFP-tagged Om45, expressing LacZ, LRRK2^{RCK}, R1398L^{RCK} or G2019S^{RCK}. Representative confocal micrographs of day 2 are shown. Dead cells were visualized via propidium iodide (PI) counterstaining. Scale bar represents 5 µm.

(B) Flow cytometric quantification of the Om45-GFP mean fluorescence intensity of cells described in **(A)**. Intensities were normalized to control cells on day 1. Dead cells were excluded from the analysis via PI counterstaining. Means ± SEM; *n*=6; *** p<0.001, n.s. not significant.

Supplementary Table S1: Plasmids and oligonucleotides used for gene disruption, chromosomal tagging, overexpression and reverse transcription quantitative PCR.

Modification	Oligonucleotides	PCR template
**Tagging and deletion of genes**		
C-terminal tagging of *OM45* with GFP	5'- ATTCAAAGAATGGAATGATAAGGGTGATGGTAAATTCTGGAG CTCGAAAAAGGACCGTACGCTGCAGGTCGAC -3'<br>5'- GAATATGTATATATGTTATGCGGGAACCAACCCTTTACAATT AGCTATCTAACTAATCGATGAATTCGAGCTCG -3'	pYM25
Control PCR *OM45*	5'- GCCAGAGGTTTAGAAGGATGGGG -3'<br>5'- GTCGACCTGCAGCGTACG -3'	
Deletion of *ATG1*	5'- ACCCCATATTTCAAATCTCTTTACAACACCAGACGAGAAAT TAAGAAAATGCGTACGCTGCAGGTCGAC -3'<br>5'- ATATAGCAGGTCATTTGTACTTAATAAGAAAACCATATTATGC ATCACTTA ATCGATGAATTCGAGCTCG -3'	pFA6a-hphNT1
Control PCR *ATG1*	5'- GTAATGTAAGGAAAACCCAC -3'<br>5'- GTCGACCTGCAGCGTACG -3'	
Deletion of *ATG11*	5'- GTGTACTGTTGTTGTTCGGAAAGTACTTCTTTTATTTTCTTTTAT ACATCATGCGTACGCTGCAGGTCGAC -3'<br>5'- GATACATAATTAAAATCTTGTCATTTGTGACAAACGTTTAGCA CTGTTCAATCGATGAATTCGAGCTCG -3'	pFA6a-hphNT1
Control PCR *ATG11*	5'- GCTAGCATTCCTATATATCC -3'<br>5'- GTCGACCTGCAGCGTACG -3'	
Deletion of *ATG32*	5'- ATTGAAGTCCTAATCACAAAAGCAAAAAAAATCTGCCAGGAAC AGTAAACATATG CGTACGCTGCAGGTCGAC -3'<br>5'- GATAGTAAAAAAGTGAGTAGGAACGTGTATGTTTGTGTATATTG GAAAAAGGTTAATCGATGAATTCGAGCTCG -3'	pFA6a-natNT2
Control PCR *ATG32*	5'- CGTACGCTGCAGGTCGAC -3'<br>5'- GATACGCAGTGAGAGAAACAGAAG -3'	
**Overexpression**		
C-terminal fusion of LRRK2^{RCK} and R1398L^{RCK} with mCherry	5'- ATATATTCTAGAATGGTTTCAAAAGGTGAAGATG -3'<br>5'- ATATATGTTTAAACCCTTATTTATATAATTCATCCATACCACC -3'	pYM27_mCherry
mCherry	5'- ATATATGGATCCATGGTTTCAAAAGGTGAAGATG -3'<br>5'- TATATGCGGCCGCCCTTATTTATATAATTCATCCATACCACC -3'	pYM27_mCherry

q-RT-PCR	
OM45	5’- AGGCTAGGGAAGAGGCTCCA -3’<br>5’- GCTTGCGTGTCTGAGCATCC -3’
MDH1	5’- GTCAATGGCCCATGCTGGTG -3’<br>5’- GGCCCAAAGTGACCGGAGAT -3’
POR1	5’- AGCAAACCGGCTTGGGTCTA -3’<br>5’- ACCAGGGGTCAAGTTGGCAA -3’
COX4	5’- AACCCGTGGTGAAAACTGCC -3’<br>5’- GGTCTGTTGGAACGGTACCCT -3’
HAP4	5’- TCGAAGTCGAACGCTAACCT -3’<br>5’- GGTCGTCGATGAAACTGCTT -3’
MSS51	5’- TCGTCACCTCATGGGGTTCG -3’<br>5’- GCGTTCTAATCTTGGCGGCC -3’
PGC1	5’- CAGTGTGCCATCCAGGAGCT -3’<br>5’- AAAGCCCCCACGTGATCCTC -3’
UBC6	5’- TGCTCGCCCCAACGAAGATA -3’<br>5’- ACCGTGATATTGACCGCCCT -3’

Supplementary Table S2: Strains used in this study.

Strain	Genotype	Source
BY4741	MAT**a**; *his3*Δ1; *leu2*Δ0; *met*15Δ0; *ura3*Δ0	Euroscarf
W303	MAT**a;** *leu2-3,112*; *trp1-1*; *can1-100*; *ura3-1*; *ade2-1*	Euroscarf
BY4741 Δ*atg1*	BY4741 *atg1*Δ::kanMX4	This study
BY4741 Δ*atg11*	BY4741 *atg11*Δ::kanMX4	This study
BY4741 Δ*atg32*	BY4741 *atg32*Δ::kanMX4	This study
BY4741 *OM45-GFP*	BY4741 *OM45-GFP*::hphNT1	This study
BY4741 *PMA1-GFP*	BY4741 *PMA1-GFP*::HIS3MX6	Euroscarf
BY4741 *HTB2-GFP*	BY4741 *HTB2-GFP*::HIS3MX6	Euroscarf
